# Comparative Pharmacovigilance Analysis of Safety Signals Among Advanced Prostate Cancer Therapies Using FAERS (FDA Adverse Event Reporting System)

**DOI:** 10.3390/curroncol33090530

**Published:** 2026-09-02

**Authors:** Zaid Ahmed, Rashid Sayyid, Omid Yazdanpanah, Ravand Samaeekia, Arash Rezazadeh Kalebasty, David I. Lee, Mohammed Shahait

**Affiliations:** 1 Department of Urology, University of California, Irvine School of Medicine, Orange, CA 92868, USA; dilee1@hs.uci.edu (D.I.L.); mshahait@hs.uci.edu (M.S.); 2 Department of Urology, College of Medicine—Tucson, University of Arizona, Tucson, AZ 85724, USA; rsayyid@arizona.edu; 3 Division of Hematology and Oncology, Department of Medicine, University of California, Irvine School of Medicine, Orange, CA 92868, USA; oyazdanp@hs.uci.edu (O.Y.); rsamaeek@hs.uci.edu (R.S.); arez@hs.uci.edu (A.R.K.)

**Keywords:** prostate cancer, pharmacovigilance, drug safety, adverse events

## Abstract

Therapeutic options for advanced prostate cancer have expanded in recent years, incorporating multiple-system treatment approaches with differing mechanisms of action. However, comparative real-world safety data following drug approval remain limited. As such, the aim of this study is to characterize adverse events and disproportionate safety signals among advanced prostate cancer therapies using the FDA Adverse Event Reporting System (FAERS). A retrospective pharmacovigilance study of FAERS reports evaluated enzalutamide, darolutamide, apalutamide, abiraterone acetate, relugolix, niraparib/abiraterone, talazoparib, rucaparib, cabazitaxel, sipuleucel-T, and lutetium-177 vipivotide. Adverse events were categorized by System Organ Class and Preferred Terms. Reporting odds ratios (RORs) with 95% confidence intervals identified safety signals. Among 172,440 reports, most involved patients aged 65–85 years. Cabazitaxel had the highest proportion of serious reports (86.6%) and deaths (22%), whereas relugolix had the lowest (23.3% and 4.8%). Nervous system disorders predominated with enzalutamide and darolutamide, gastrointestinal disorders with abiraterone, rucaparib, and niraparib/abiraterone, and hematologic toxicities with cabazitaxel, talazoparib, and lutetium-177 vipivotide. Significant safety signals were identified for abiraterone and cabazitaxel, but not other therapies. The absence of a detected signal should not be interpreted as evidence of safety or equivalence, as reporting volume, detection bias, and statistical power varied across therapies. Overall, the therapies demonstrated distinct toxicity profiles, which may inform treatment selection, toxicity monitoring, and patient counseling.

## Simple Summary

Advanced prostate cancer is treated with several types of medications, including hormone therapies, chemotherapy, targeted therapies, immunotherapy, and radioligand therapy. Although these treatments have been proven effective in clinical trials, less is known about how they perform in routine clinical practice, where larger and more diverse patient populations may experience uncommon side effects. In this study, more than 172,000 reports from the U.S. Food and Drug Administration Adverse Event Reporting System were analyzed to compare the safety profiles of commonly used advanced prostate cancer therapies. The findings suggest that different treatments were associated with distinct patterns of side effects. Chemotherapy was linked to the greatest proportion of serious adverse events, whereas hormonal therapies and other targeted treatments showed more organ-specific toxicity patterns. These findings provide real-world evidence that may help clinicians better anticipate treatment-related toxicities, improve patient counseling, and guide safer treatment selection for men with advanced prostate cancer.

## 1. Introduction

Advanced prostate cancer remains a leading cause of cancer-related morbidity and mortality among men worldwide. Prostate cancer is the most common cancer among men in the United States, and the incidence of advanced disease is increasing rapidly, with distant-stage disease increasing by 6.0% annually in men aged 55–69 years and 6.2% annually in men aged 70 years and older [1].

Over the past decade, the therapeutic landscape has expanded substantially with the introduction of multiple systemic treatment classes, including hormonal therapies, poly (ADP-ribose) polymerase (PARP) inhibitors, radioligand therapies, immunotherapy, and cytotoxic chemotherapy [2,3]. Treatment selection is increasingly shaped by mechanisms of resistance and biomarker-guided strategies, as both androgen receptor-dependent and independent pathways can influence responses to systemic therapies [4]. While these advancements have extended overall survival and improved quality of life, the increasing complexity and heterogeneity of available therapies present new challenges in clinical practice, particularly in the identification, monitoring, and management of treatment-related toxicities [5].

Prior studies have characterized the safety profiles of individual agents or specific drug classes, primarily through randomized controlled trials (RCTs), pooled safety analyses, and systematic reviews [6,7]. Additionally, select real-world and pharmacovigilance studies have evaluated adverse events associated with individual therapies, such as androgen receptor inhibitors or PARP inhibitors, demonstrating class-specific toxicities including cardiovascular, neurologic, and hematologic complications [8]. While RCTs provide insight into drug efficacy and safety, they are often conducted in highly selected patient populations under controlled conditions, which may limit generalizability to real-world settings. As a result, uncommon, delayed, or population-specific adverse events may be underrepresented or not fully captured [9].

Pharmacovigilance databases, such as the U.S. Food and Drug Administration Adverse Event Reporting System (FAERS), offer valuable insights into real-world safety profiles across diverse patient populations and therapeutic classes. By utilizing large-scale reporting data, pharmacovigilance analyses can identify disproportionate safety signals and characterize adverse event patterns not present in clinical trials. These insights are particularly important in advanced prostate cancer, where patients are often older, have multiple comorbidities, and may receive sequential or combination therapies that further complicate toxicity profiles.

With the rise of novel hormonal agents, targeted therapies, and cytotoxic regimens, there remains a need for comprehensive and comparative safety assessments across treatment classes. This study aims to characterize adverse event patterns and identify disproportionate safety signals among commonly used therapies for advanced prostate cancer by performing a large-scale pharmacovigilance analysis using FAERS.

## 2. Materials and Methods

A retrospective pharmacovigilance study was conducted using the U.S. Food and Drug Administration Adverse Event Reporting System (FAERS). The database provides spontaneous reports of adverse drug events submitted by healthcare professionals, manufacturers, and consumers. FAERS data were analyzed from 1 January 2010 through 31 December 2025.

FAERS reports for therapies used in the treatment of advanced prostate cancer were identified using both generic and brand names. Evaluated agents included androgen receptor pathway inhibitors (enzalutamide, apalutamide, darolutamide, abiraterone acetate), gonadotropin-releasing hormone (GnRH) antagonist (relugolix), PARP inhibitors (talazoparib, rucaparib), combination therapy (niraparib plus abiraterone acetate), cytotoxic chemotherapy (cabazitaxel), immunotherapy (sipuleucel-T), and radioligand therapy (lutetium-177 vipivotide). For each therapy, total case counts, proportions of serious adverse events, and proportions of death reports were calculated. Duplicate reports were identified and removed in accordance with FDA-recommended deduplication practices using case identifiers and report version numbers. Reports were analyzed irrespective of reporter type (healthcare professional, consumer, or manufacturer). Drugs listed as primary or secondary suspected agents were included to maximize signal detection. Combination products, including niraparib plus abiraterone (Akeega), were analyzed as individual therapeutic entities according to the reported product name in FAERS.

Adverse events were coded using the Medical Dictionary for Regulatory Activities (MedDRA) terminology and categorized at both the System Organ Class (SOC) and Preferred Term (PT) levels. Analyzed outcomes included total adverse event reports, serious adverse events and death reports. Serious adverse events were defined by FAERS to include death, life-threatening events, hospitalization, disability, or other medically significant outcomes. Additional analyses included age distribution of reported cases (18–64, 65–85, >85 years, and unspecified), characterization of dominant SOC categories for each therapy, and comparison of reporting patterns by brand versus generic drug names. Descriptive analyses are presented as counts and percentages. SOC-level distributions were used to characterize broad toxicity patterns across therapies, while PT-level analyses were used to identify specific adverse events.

Disproportionality analysis was performed using reporting odds ratios (RORs) with corresponding 95% confidence intervals (CIs) to identify potential safety signals. RORs were calculated by comparing the odds of a specific adverse event for a given drug relative to all other drugs in the database. For each evaluated drug–PT combination, a two-sided Fisher’s exact test was performed using the corresponding 2 × 2 contingency table. To account for multiple comparisons, *p*-values were adjusted using the Benjamini–Hochberg procedure across all drug-PT combinations. A PT-level signal was defined as an association supported by at least three drug–event reports, a lower bound of the ROR 95% confidence interval greater than 1.0, and an FDR-adjusted *q*-value < 0.05. A minimum of three reports was used as an exploratory signal detection threshold, with report counts and confidence intervals used to support signal strength and accuracy. Analyses were conducted at the PT level to identify specific adverse event signals and at the SOC level to characterize broader toxicity patterns. Statistical analyses were performed using R software (version 4.5.2; R Foundation for Statistical Computing, Vienna, Austria).

## 3. Results

A total of 172,440 FAERS reports were identified across the advanced prostate cancer therapies examined. Across all therapies, there was notable variation in the proportion of serious adverse events and death outcomes observed (Table 1). Cabazitaxel demonstrated the highest proportion of serious reports (86.6%) and deaths (22.0%), reflecting its prominent cytotoxic toxicity profile. On the contrary, relugolix had the lowest proportion of serious events (23.3%) and deaths (4.8%). Intermediate levels of serious and fatal cases were observed for the remaining androgen receptor pathway inhibitors and PARP inhibitors.

An analysis of age distribution found that patients aged 65–85 years accounted for the largest proportion of FAERS reports across nearly all therapies (Table 2). Specific therapies, including PARP inhibitors and combination regimens, demonstrated a higher proportion of reports in younger age groups. Across most therapies, the reports did not include the age of patients and remained not specified in the analysis.

**Figure 1:**
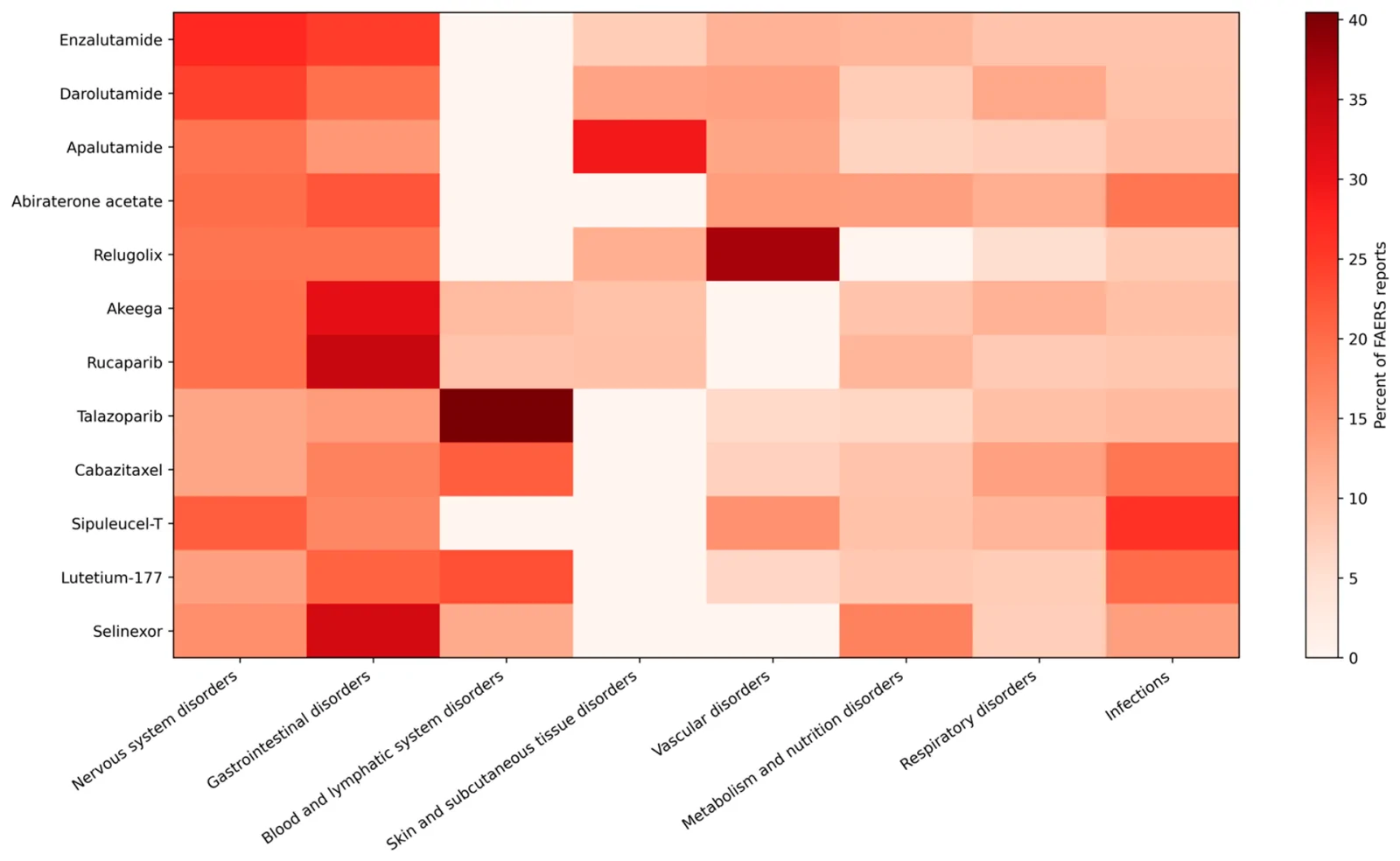
Distribution of Adverse Event Reports by System Organ Class Across Advanced Prostate Cancer Therapies.

Analysis of adverse events by System Organ Class (SOC) depicted distinct toxicity patterns across the therapies examined (Figure 1). Nervous system disorders were the most prominent outcomes for enzalutamide, darolutamide, and Sipuleucel-T. Gastrointestinal disorders predominated for abiraterone acetate, rucaparib, and Akeega. Hematologic and lymphatic system disorders were most evident among cabazitaxel, talazoparib, and lutetium-177 vipivotide. Apalutamide was associated with a higher proportion of skin and subcutaneous tissue disorders, while relugolix demonstrated a predominance of vascular-related events.

Following Benjamini–Hochberg correction across all evaluated drug–PT combinations, disproportionality analysis identified five statistically significant Preferred Term (PT)-level signals for abiraterone acetate and 16 for cabazitaxel (Table 3), all with FDR-adjusted *q*-values < 0.001. For abiraterone acetate, the identified signals were increased alanine aminotransferase (ROR 4.34, 95% CI 3.83–4.91), hypertension (ROR 3.12, 95% CI 2.90–3.36), bone pain (ROR 3.00, 95% CI 2.60–3.46), fatigue (ROR 2.32, 95% CI 2.22–2.43), and back pain (ROR 1.42, 95% CI 1.28–1.57). The strongest cabazitaxel signals included sudden death (ROR 11.93, 95% CI 6.42–22.17), thrombocytopenia (ROR 11.11, 95% CI 9.19–13.43), and sepsis (ROR 10.64, 95% CI 8.77–12.90). No PT-level disproportionality signals satisfying the prespecified criteria were identified for the remaining nine therapies.

## 4. Discussion

In this large, real-world pharmacovigilance analysis of 172,440 FAERS reports spanning 2010–2025, distinct and clinically meaningful differences in adverse event profiles were identified across the major therapeutic classes used in advanced prostate cancer. With distinct findings across SOC-level toxicity patterns and class-specific toxicities, these findings provide a comprehensive, comparative assessment of multiple therapeutic modalities. As with all spontaneous reporting databases, these findings should be interpreted cautiously, as FAERS is subject to underreporting, reporting bias, variable data quality, and an inability to establish causality or estimate true incidence rates. Nevertheless, pharmacovigilance analyses remain valuable for identifying potential safety signals that may complement evidence generated from clinical trials.

At the class level, cytotoxic chemotherapy demonstrated the highest burden of severe and fatal adverse events. Cabazitaxel exhibited the highest proportion of serious outcomes and the greatest number of statistically significant PT-level safety signals, reflecting its established hematologic and systemic toxicity profile. These findings are consistent with the established safety profile observed in the pivotal TROPIC phase III trial, which reported high rates of grade 3–4 neutropenia, febrile neutropenia, and diarrhea among patients receiving cabazitaxel [10]. Beyond toxicity, response to taxane therapy may also be influenced by the tumor microenvironment and molecular mechanisms of resistance, as identified by Peng et al. [11]. However, the current analysis identified disproportionate reporting of sudden death and multi-organ failure, events that were uncommon in clinical trials. This discrepancy likely reflects cabazitaxel general being administered in later treatment lines, whereas relugolix and other androgen receptor pathway inhibitors are frequently introduced earlier in the treatment course [12]. Patients have shown shorter survival and greater comorbidity burden among cabazitaxel-treated patients, indicating alternative sources as better predictors of mortality.

In contrast, androgen receptor pathway inhibitors demonstrated more organ-specific toxicity patterns, with nervous system disorders predominating among enzalutamide and darolutamide. These findings are consistent with the safety profiles reported in pivotal phase III trials of enzalutamide, including PREVAIL and AFFIRM, which identified fatigue, falls, and neurologic adverse events among the most common treatment-related toxicities [13,14]. Although darolutamide has demonstrated lower blood–brain barrier penetration and a favorable central nervous system safety profile in clinical trials, nervous system disorders remained the predominant System Organ Class reported in FAERS [15,16]. This observation may reflect differences between carefully selected trial populations and the broader, older, and more medically complex patients encountered in routine clinical practice. However, age was unspecified in 33.5% of darolutamide reports, in addition to FAERS not containing comorbidity data. Furthermore, channeling bias amongst providers may provide another explanation, as darolutamide may be preferentially selected due to its favorable CNS profile. Consequently, the observed SOC distribution should not be interpreted as evidence that darolutamide causes greater neurologic toxicity.

Gastrointestinal toxicities were most prominent among abiraterone acetate and PARP inhibitor-based therapies, including rucaparib and niraparib plus abiraterone. These findings are consistent with the safety profiles reported in pivotal trials of abiraterone and PARP inhibitor combination therapy, in which gastrointestinal adverse events and hepatic enzyme elevations were among the most reported treatment-related toxicities [17,18,19]. The disproportionate reporting of alanine aminotransferase elevation observed with abiraterone in our analysis further supports its established hepatotoxicity profile. These findings reinforce the importance of routine laboratory monitoring and early recognition of gastrointestinal and hepatic toxicities to optimize treatment tolerability in clinical practice.

Hematologic and lymphatic system disorders were also prominent among cabazitaxel, talazoparib, and lutetium-177 vipivotide. The high frequency of hematologic adverse events observed with cabazitaxel is consistent with its established myelosuppressive profile as a taxane chemotherapy, including risks of neutropenia, anemia, and infection-related complications [20,21,22]. Similarly, PARP inhibitors such as talazoparib and radioligand therapy have been associated with the impairment DNA repair and bone marrow toxicity, contributing to anemia and thrombocytopenia [23,24,25].

Apalutamide demonstrated a predominance of skin and subcutaneous tissue disorders, consistent with previous findings such as the SPARTAN trial, which found higher rates of rash compared to placebo [26,27,28]. However, no individual skin-related PT met the adjusted disproportionality criteria. Similarly, the predominance of vascular disorders among relugolix reports did not translate into any significant PT-level signals. In studies, including the HERO trial, major adverse cardiovascular events occurred less frequently with relugolix than leuprolide, further emphasizing that SOC reporting portions should not be interpreted as direct, comparative evidence [29,30].

From a clinical standpoint, these SOC-level differences have crucial implications for treatment selection and toxicity monitoring for patients. Given that patients with advanced prostate cancer are typically older and have a higher burden of comorbid conditions, they may be more susceptible to treatment-related adverse events. Consequently, the findings of this study highlight the importance of toxicity surveillance and individualized risk stratification, allowing providers to tailor therapy selection and monitoring strategies to minimize adverse outcomes.

Future research should validate these findings using prospective and longitudinal studies to better establish causal relationships between specific therapies and adverse event profiles. The integration of clinical variables such as existing comorbidities, prior treatment exposure, disease severity, and the combination of multiple therapies are critical in defining the risk and toxicity patterns observed in this study. Recent findings have examined the role of molecular characteristics such as aneuploidy-associated driver genes and the role in the progression and metastasis of prostate cancer. Despite these factors not being captured in FAERS, they may influence future decisions regarding treatment selections [31]. Finally, studies exploring the biological basis of observed SOC-level toxicities may help identify predictive biomarkers and guide more personalized approaches to therapy selection and toxicity mitigation.

This study has several limitations inherent to spontaneous pharmacovigilance databases. FAERS is subject to underreporting, reporting bias, incomplete clinical information, duplicate reporting despite standard deduplication procedures, and the inability to establish causality or estimate adverse event incidence. These limitations should be considered when interpreting the reported safety signals. Furthermore, including both primary and secondary suspect agents increased sensitivity for exploratory signal detection, but may have introduced noise and exposure misclassification. Reported events introduce the potential of concomitant therapies, underlying malignancy, or disease progression among the adverse events. Additionally, confounding by indication, concomitant therapies and medications, and patient comorbidities cannot be fully accounted for using the database. There was a high proportion of not specified data accounting for demographics and age limit subgroup analysis, as well as generalizability across therapies. This is concurrent with the absence of statistically significant disproportionality signals for several therapies, such as for lutetium-177 vipivotide, which had the highest proportion of unspecified age reports. Consequently, demographic comparisons involving this therapy should be interpreted cautiously. A lack of a signal does not fully imply absence of risk but may reflect underreporting and the absence of longitudinal data from FAERS. Lower report counts and absent signals for newer therapies may also reflect shorter post-marketing periods, lower market penetration, and less cumulative real-world exposure rather than safer toxicity profiles.

## 5. Conclusions

In this pharmacovigilance analysis, the findings present a comprehensive and comparative safety profile across advanced prostate cancer therapies. Meaningful differences in toxicity patterns and adverse events may inform clinical decision-making, while considering that the absence of a detected signal does not establish safety equivalence and may reflect limited reporting or statistical power. Due to the high personalization of treatment selection, integrating pharmacovigilance data alongside existing clinical trial evidence will be essential to optimize therapeutic outcomes.

## Figures and Tables

**Table 1:** Overview of FAERS Reports for Advanced Prostate Cancer Therapies.

Drug	Total Cases	% Serious Cases	% Death	Major SOC (%)
Enzalutamide (Xtandi)	53,958	61.6%	20.1%	Nervous system (51.9%)
Darolutamide (Nubeqa)	3664	78.9%	14.9%	Nervous system (41.9%)
Apalutamide (Erleada)	10,008	63.5%	13.3%	Skin and subcutaneous tissue (36.9%)
Abiraterone acetate (Zytiga)	35,361	66.7%	21.2%	Gastrointestinal (11.1%)
Relugolix (Orgovyx)	21,779	23.3%	4.8%	Vascular (42.1%)
Akeega (Niraparib + Abiraterone)	8448	82.0%	9.0%	Gastrointestinal (18.9%)
Talazoparib (Talzenna)	1581	80.7%	18.3%	Blood and lymphatic system (42.0%)
Rucaparib (Rubraca)	8701	43.2%	5.8%	Gastrointestinal (31.1%)
Cabazitaxel (Jevtana)	3103	86.6%	22.0%	Blood and lymphatic system (35.6%)
Sipuleucel-T (Provenge)	5850	48.1%	6.6%	Nervous system (16.3%)
Lutetium Lu 177	12,192	25.2%	13.0%	Blood and lymphatic system (24.2%)

**Table 2:** Age Distribution of FAERS Reports by Advanced Prostate Cancer Therapy.

Drug	18–64 Years Old	65–85 Years Old	>85 Years Old	Not Specified
Enzalutamide	7.9%	42.0%	9.4%	40.7%
Darolutamide	12.1%	45.9%	8.5%	33.5%
Apalutamide	5.8%	40.9%	8.4%	44.9%
Abiraterone acetate	8.7%	41.5%	7.5%	42.2%
Relugolix	6.2%	26.0%	2.9%	64.9%
Akeega	39.2%	51.6%	2.9%	6.3%
Talazoparib	34.3%	36.2%	1.3%	26.5%
Rucaparib	19.9%	21.3%	0.8%	58.0%
Cabazitaxel	19.2%	49.0%	1.1%	30.6%
Sipuleucel-T	9.2%	37.8%	3.9%	49.1%
Lutetium Lu 177	1.4%	6.4%	0.5%	91.6%

**Table 3:** Preferred Term (PT) Safety Signals Identified by Disproportionality Analysis.

Drug	Preferred Term	ROR	95% CI	FDR-Adjusted *q*-Value
Abiraterone acetate	Back pain	1.42	1.28–1.57	<0.001
	Alanine aminotransferase increase	4.34	3.83–4.91	<0.001
	Bone pain	3.00	2.60–3.46	<0.001
	Fatigue	2.32	2.22–2.43	<0.001
	Hypertension	3.12	2.90–3.36	<0.001
Cabazitaxel	Sudden death	11.93	6.42–22.17	<0.001
	Gastrointestinal hemorrhage	3.56	2.47–5.13	<0.001
	Multi-organ failure	7.16	4.96–10.32	<0.001
	Febrile neutropenia	8.51	6.03–9.62	<0.001
	Anemia	8.90	7.57–10.47	<0.001
	Thrombocytopenia	11.11	9.19–13.43	<0.001
	Sepsis	10.64	8.77–12.90	<0.001
	Pneumonia	3.30	2.71–4.00	<0.001
	Diarrhea	3.09	2.66–3.59	<0.001
	Nausea	1.50	1.24–1.81	<0.001
	Fatigue	2.41	2.07–2.80	<0.001
	Asthenia	3.60	3.00–4.30	<0.001
	Dyspnoea	2.55	2.15–3.03	<0.001
	Haematuria	3.99	3.24–4.68	<0.001
	Decreased appetite	4.40	3.60–5.39	<0.001
	Pyrexia	5.33	4.56–6.24	<0.001

## Data Availability

The data that support the findings of this study are available from the corresponding author upon reasonable request.
